# Evolution of radical mastectomy for breast cancer

**Published:** 2016

**Authors:** M Plesca, C Bordea, B El Houcheimi, E Ichim, A Blidaru

**Affiliations:** * 2nd Department of Oncological Surgery, Oncological Institute, Bucharest, Romania

**Keywords:** radical mastectomy, breast cancer

## Abstract

Surgical treatment of breast cancer has been marked by a constant evolution since the Halsted radical mastectomy described in the late 19th century has become the current standard Madden radical mastectomy, a breast surgery that involves the ablation of tissue with the axillary lymphatic preserving both pectoral muscles. The purpose of this paper was to present the stages that have marked the evolution of this intervention and to provide an overview of the way breast cancer has been understood and treated in the last century.

## Introduction

The current standard in radical mastectomy was established by John Madden in 1972 [**1**]. His contribution to the technique was the preservation of both pectoral muscles. Between 1894, when William Halsted performed the surgery that bears his name [**2**], and the modern era in the surgical treatment of breast cancer, there have been attempts to expand the scale of the intervention, but these operations, called supraradical mastectomies were associated with significant morbidity and proved to be of little therapeutic benefit [**3**]. After the implementation of the Madden modified radical mastectomy, along with advances made in adjuvant therapy and radiotherapy, conservative treatment was adopted because, it was shown that that it achieved similar results to mastectomy in terms of oncological safety, but with obvious benefits in terms of aesthetics [**4**]. Sentinel node technique favored the limitation of unnecessary interventions regarding axillary lymphadenectomy, and, in this way, the rate of postoperative complications (lymphedema, paresthesia, upper limb mobility limitation) was decreased considerably [**5**]. The combination of plastic surgery principles in cancer surgery gave birth to oncoplastic surgery that brought a great contribution regarding the obtaining of an optimal esthetic result, with techniques such as nipple areola sparring mastectomy or skin-sparing mastectomy, to facilitate breast reconstruction [**6**]. Although controversial in terms of cost and learning curve, the use of robotic surgery in breast surgery, particularly in skin sparring mastectomy can be a solution because of the advantages that robotic dissection offers [**7**].

**Halsted’s Mastectomy**

There is no reliable data on the origin of mastectomy but it is known that it was practiced routinely in breast cancer patients since the days of the Byzantine Empire [**8**]. In 1882, William Halsted documented the first interventions he carried out, establishing guidelines in radical cancer surgery and using new anesthesia, aseptic and antiseptic techniques for the first time. Results in terms of survival and local recurrence reduction were exceptional, thus making the Halsted operation, described in the 19th century, be performed on more than 90% of the patients with breast cancer in the US until the 1970s of the 20th century [**9**]. Halsted’s radical mastectomy involved large incisions and extensive tissue ablation. The mammary gland, both pectoral muscles, and the entire axillary lymphatic tissue, up to its tip, were excised. The advantage of the technique is the facilitation of access to the axillary vein, which can be completely denuded [**10**]. The extent of resection also led to an important associated morbidity (paraesthesia, lymphedema of the arm, rib cartilage damage, or pneumothorax by the perforation of the intercostal space). The hypothesis on the futility of such radical an intervention was initially advanced by Haagensen in 1935, but was confirmed by Bernard Fisher in 1971 with the publication of the results of the first prospective study comparing Halsted mastectomy to modified radical mastectomy that preserves pectoral muscles, with comparable results in terms of survival [**11**]. David H. Patey modified Halsted’s operation by keeping the great pectoral muscle. The surgery is less traumatic and is followed by less postoperative complications (axillary retractable scar, painful syndrome, lymphedema, upper limb mobility limitation). Lymphedema was not constant and the postoperative outcome was better with the preservation of the great pectoral and by changing the type of incision, which was oblique or transverse, and circumscribed the breast as an ellipse with poles on the xiphoid medial breast and axillary lateral basis [**12**].

**Madden modified radical mastectomy**

The current standard for mastectomy is the surgery described by Madden in 1972. He concluded that keeping both pectoral muscles gives the best result. Madden mastectomy involves making an elliptical incision circumscribing the breast and including the nipple areola complex while having as a central landmark the site of the tumor. Thus, for a tumor located in the lower quadrants of the breast, the upper limit of the incision will be just above the areola while the lower limit will be placed towards the inframammary fold, to allow the inclusion of as much tissue close to the tumor site as possible. The mammary gland is separated from the skin flaps by cutting the Cooper’s ligaments. Mammary gland ablation is done simultaneous with the pectoralis major fascia to reduce the risk of chest wall recurrence, although some authors disagree with this aspect particularly for early stage cases. Axillary lymphadenectomy is a mandatory component of radical mastectomy. The following stations are targeted: brachial lymph node group (lateral), pectoral lymph node groups (superior), subscapular lymph node groups (posterior), central nodal group, and apical lymph node group (medial or subclavicular). In need of a surgical systematization, John W. Berg divided the axillary lymph nodes into three stations according to the position they occupied in relation to the minor pectoris muscle. The first station comprised nodes located outside the external edge of the minor pectoris muscle. A second station included nodes that were found behind it and the third station was contained in a space within the internal edge of the muscle. Lower axillary lymphadenectomy involves the ablation of the first station, while complete axillary lymphadenectomy means that all three stations are taken out [**13**].

A controversy on Madden’s mastectomy concerned the interpectoral lymphatic group (Rotter’s group). In a prospective study on a group of 172 patients, published in 2005, in Tumori, Vrdoljak concluded that up to 30% of the patients with axillary lymph node invasion need interpectoral lymph node ablation [**14**]. A similar proportion has been indicated by a study conducted by Gregory earlier [**15**]. The author recommended a routine nodal ablation because the surgical approach of this group was easy and the aesthetic result was not affected. However, the pedicle of the pectoral can produce damage at this level, which could also mean that the great pectoral muscle could develop a partial atrophy [**16**]. In some cases, axillary lymphadenectomy leads to a serious complication, lymphedema. Once installed, the treatment has only a palliative role. For this reason, many tried to minimize the extent of dissections without harming the surgical safety. Thus, there were authors, including Benson and Procaccini, who issued theories on the required number of lymph nodes for a correct histological examination. Thus, initially 10 nodes were thought to be enough, then 4 [**17**]. All these dilemmas were answered with the introduction of the sentinel node technique, the procedure that identifies the first lymph node to which the tumor drains lymph. Through high sensitivity and specificity of the method, practically unnecessary resections are excluded [**18**].

**Fig. 1:**
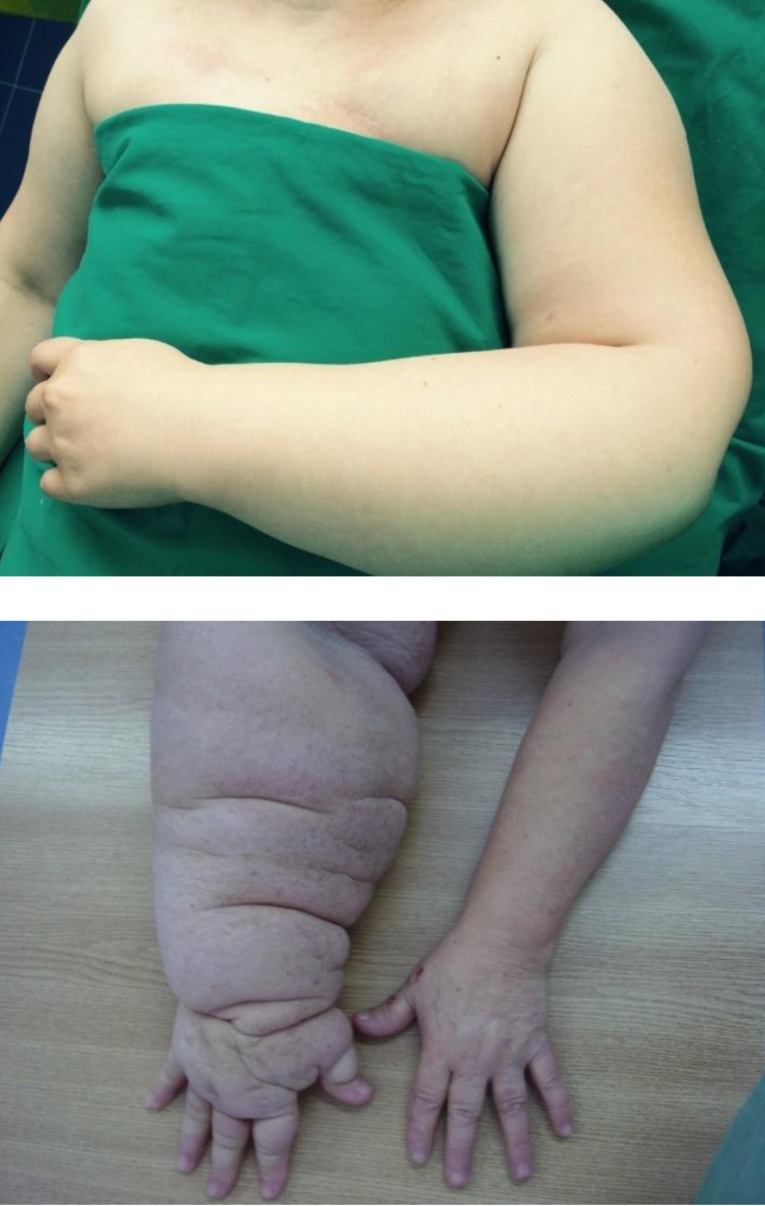
Postmastectomy lymphedema of the arm

**Fig. 2:**
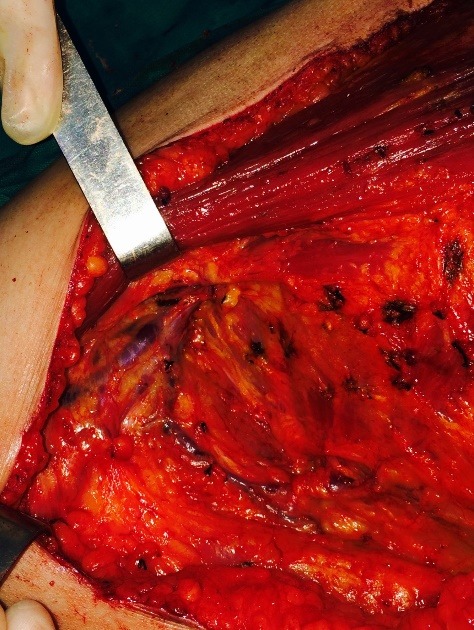
Madden Mastectomy – Complete axillary

**The Romanian experience**

Romanian physicians had their own contribution to breast cancer surgery, with names like Ion Chiricuta or Alexandru Trestioreanu. By analogy with Chimera, a character from Greek mythology composed of segments belonging to different animals, Chiricuta proposed a “chimerization” of the pectoral muscles. The intervention did not make concessions regarding radicalism, both axillary and interpectoral lymph nodes being excised. It comes instead with an artifice, which consists of removing parts of the pectoral muscles to ensure the easy access for performing extensive axillary lymphadenectomy and suturing the remaining part of the muscles afterwards, thus creating a “muscular screen” as Trestioreanu described this surgery in 1978 at a scientific session of the Oncology Institute in Cluj [**19**].

With the advance of imaging and radiology, the detection of suspicious breast lesions has been achieved from non-palpable stages; thus, conservative treatment has emerged as a viable alternative. In order to operate on such small lesions, a preoperative localization is required, usually by ultrasound or mammography in case of suspicious microcalcifications. Conservative treatment for breast cancer is not only addressed in early stage cases but also for tumors that respond well to neoadjuvant therapy and have a less aggressive histopathological and immunohistochemical profile. The placement of radiopaque markers in the tumor bed when the biopsy is performed to guide the subsequent surgery is appropriate. In case of ptosis, oncoplastic surgery techniques, which can address both breasts to achieve, for example symmetrization, can produce a favorable postoperative outcome in terms of aesthetics, which may often matter for the patient’s evolution. Currently, all aspects of the multimodal treatment of breast cancer are covered, from diagnosis to treatment and follow-up, at the Bucharest Institute of Oncology. 

**Perspectives**

Breast cancer is a disease with a pronounced impact in society. A mastectomy is mutilating for a woman. The doctor who treats breast cancer is quite often put in difficulty by the disarming progression of the disease, despite all efforts. Perhaps for this reason, the approach on breast cancer was radical over time. The limits of radicalism have sometimes been pushed beyond a net benefit for the patient. The argument of this approach was the sacrificing of the quality of life to ensure survival. However, history has shown that, in many cases, this approach would have been different. Thus, conservative treatment has now reached from Halsted’s mastectomy. It is possible that future clinical trials, currently in progress, will confirm the hypothesis set forth by the American College of Oncologists and Surgical Oncologists (ACOSOG Z0011), which demonstrated that the extension of axillary lymphadenectomy does not significantly influence survival [**20**]. Before these conclusions are implemented in daily practice, further studies are needed to confirm and validate the results obtained so far.

**Acknowledgements**

This work received financial support through the project entitled “CERO- Career profile: Romanian Researcher”, grant number POSDRU/159/1.5/S/135760, cofinanced by the European Social Fund for Sectoral Operational Programme Human Resources Development 2007-2013.

## References

[1] Madden JL, Kandalaft S, Bourque RA (1972). Modified radical mastectomy. Ann Surg.

[2] Halsted WS (1907). The results of radical operations for the cure of cancer of the breast. Trans Am Surg Assoc.

[3] Urban JA (1964). Surgical excision of internal mammary nodes for breast cancer. Br J Surg.

[4] Veronesi U (1977). Conservative treatment of breast cancer: a trail in progress at the Cancer Institute of Milan. World J Surg.

[5] Giuliano AE, Kirgan DM, Guenther JM (1994). Lymphatic mapping and sentinel lymphadenectomy for breast cancer. Ann Surg.

[6] Gerber B, Krause A, Dieterich M (2009). The oncological safety of skin sparing mastectomy with conservation of the nipple-areola complex and autologous reconstruction: an extended follow-up study. Ann Surg.

[7] Toesca  A, Peradze  N, Galimberti  V, Manconi  A, Intra  M, Gentilini  O, Sances  D, Negri  D, Veronesi  G, Rietjens  M, Zurrida  S, Luini  A, Veronesi  U, Veronesi  P (2015). Robotic Nipple-sparing Mastectomy and Immediate Breast Reconstruction with Implant: First Report of Surgical Technique. Ann Surg.

[8] Browning RJ, Gorgias  T (2003).

[9] National Institutes of Health Consensus Development Panel Consensus Statement (1992). Treatment of early-stage breast cancer. J Natl Cancer Inst Monogr.

[10] Halsted  WS (1894–1895). The results of operations for the cure of cancer of the breast performed at the Johns Hopkins Hospital from June 1889 to January 1894. Johns Hopkins Hospital Reports.

[11] Fisher  B (1977). United States trials of conservative surgery. World J Surg.

[12] Patey  DH, Dyson  WH (1948). The prognosis of carcinoma of the breast in relation to the type of operation performed. Br J Cancer.

[13] Berg JW (1984). Clinical implications of risk factors for breast cancer. Cancer Supplement: American Cancer Society National Conference Breast Cancer 1983.

[14] Vrdoljak  DV, Ramljak  V, Muzina  D, Sarceviç  B, Knezević  F, Juzbasić  S (2005). Analysis of metastatic involvement of interpectoral (Rotter’s) lymph nodes related to tumor location, size, grade and hormone receptor status in breast cancer. Tumori.

[15] Senofsky  GM, Moffat  FL Jr., Davis  K, Masri  MM, Clark  KC, Robinson  DS, Sabates  B, Ketcham  AS (1991). Total Axillary Lymphadenectomy in the Management of Breast Cancer. Arch Surg.

[16] Blidaru  A, Bordea  C, Voinea  S, Condrea  I, Albert  P, Houcheimi  B Rezultatele protocolului de validare a tehnicii de identificare si biopsie a ganglionului santinela in cancerul glandei mamare folosind trasor radioactiv la Institutul Oncologic Bucuresti. Chirurgia.

[17] George  R, Quan  ML, McCready  D (2009). Sentinel Lymph node biopsy in early stage breast cancer. Ontario Cancer care Program in evidence-based care.

[18] Krag  DN, Weaver DL, Alex JC, Fairbank JT (1993). Surgical resection and radiolocalization of the sentinel lymph node in breast cancer using a gamma probe. Surg Oncol.

[19] Chiricuta  I, Setlacec  D, Sarbu  P (1981). Chirurgia Ginecologica.

[20] Giuliano  AE, Haigh  PI, Brennan  MB, Hansen  NM, Kelley  MC, Ye  W, Glass  EC, Turner  RR (2000). Prospective observational study of sentinel lymphadenectomy without further axillary dissection in patients with sentinel node-negative breast cancer. J Clin Oncol.

